# The association between *ACTB* methylation in peripheral blood and coronary heart disease in a case-control study

**DOI:** 10.3389/fcvm.2022.972566

**Published:** 2022-08-18

**Authors:** Jialie Jin, Chao Zhu, Jinxin Wang, Xiaojing Zhao, Rongxi Yang

**Affiliations:** ^1^Department of Epidemiology and Biostatistics, School of Public Health, Nanjing Medical University, Nanjing, China; ^2^Department of Cardiology, Beijing Friendship Hospital, Capital Medical University, Beijing, China; ^3^Department of Cardiology, The Second Medical Centre, Chinese PLA General Hospital, Beijing, China; ^4^Military Translational Medicine Lab, Medical Innovation Research Division, Chinese PLA General Hospital, Beijing, China; ^5^Beijing Key Laboratory of Chronic Heart Failure Precision Medicine, Medical Innovation Research Division, Chinese PLA General Hospital, Beijing, China

**Keywords:** DNA methylation, ACTB gene, coronary heart disease, peripheral blood, risk assessment

## Abstract

**Background:**

Coronary heart disease (CHD) brings a heavy burden to society worldwide. Novel and minimally invasive biomarkers for the risk evaluation of CHD are urgently needed. Previous study has revealed that blood-based hypomethylation of β-actin (*ACTB*) was associated with increased risk of stroke, but not reported in CHD yet.

**Objectives:**

We aimed to explore the association between blood-based *ACTB* methylation and the risk of CHD in a case-control study in the Chinese population.

**Methods:**

The methylation level of *ACTB* was quantitatively determined by mass spectrometry in 281 CHD patients and 272 controls. The association between *ACTB* methylation and CHD risk was estimated by logistic regression analyses adjusted for possible confounding effects.

**Results:**

We found a significant association between hypermethylation of *ACTB* in peripheral blood and increased risk of CHD (odds ratios (ORs) per +10% methylation: 1.19–1.45, *p* < 0.013 for nine out of thirteen CpG sites), especially in male subjects and heart failure (HF) patients (ORs per +10% methylation: 1.20–1.43, 1.38–1.46; *p* < 0.030, 1.52 × 10^−4^, respectively). Hypermethylation of ACTB_CpG_2.3, ACTB_CpG_7.8, and ACTB_CpG_9.10 was observed in the CHD patients with minor to medium cardiac function impairment (NYHA I&II CHD cases) (ORs per +10% methylation: 1.38–1.44; *p* < 0.001). The combination of ACTB_CpG_2.3, ACTB_CpG_7.8, and ACTB_CpG_9.10 methylation levels could efficiently discriminate CHD cases, male CHD patients, HF and NYHA I&II CHD patients from controls (area under curve (AUC) = 0.75, 0.74, 0.73, and 0.77, respectively).

**Conclusions:**

Our study reveals a strong association between blood-based *ACTB* hypermethylation and CHD risk. The combination of *ACTB* methylation and conventional risk factors might provide a novel strategy to improve risk assessment of CHD.

## Introduction

Cardiovascular diseases (CVDs) are the leading cause of mortality and morbidity worldwide. The prevalent cases of total CVDs reached 523 million in 2019, and the number of CVDs deaths increased from 12.1 million in 1990 to 18.6 million in 2019 (1, 2). Coronary heart disease (CHD) is the largest contributor to CVDs. CHD is usually caused by atherosclerosis, a chronic inflammatory condition of the coronary arterial wall, and doesn't always have symptoms in the early stages of the disease (3, 4). Risk assessment employing risk factors, such as genetic background, environmental and lifestyle factors (e.g., cigarette smoking, alcohol drinking), hypertension, diabetes, and hyperlipidemia, can be used to identify individuals at high risk of CHD. However, a large number of asymptomatic individuals remain undiagnosed in primary care settings (5–7). Thus, studies on the novel risk factors of CHD are urgently needed. Genome-wide association studies (GWAS) have identified over 160 genetic loci associated with CHD risk that have genome-wide significance and over 300 additional loci suggestive for CHD risk. These loci are mostly associated with modest increases in CHD risk, and explain about 30–40% of CHD heritability, accounting for approximately 40% of all cases (8). On the other hand, recent studies have revealed DNA methylation signatures associated with the development of CHD, indicating that DNA methylation may be useful to improve the prediction of CHD risk (9). Moreover, interactions between genes and environment are implicated in pathological conditions (10, 11). Epigenetic mechanisms play a key role in the regulation of gene expression that can be modified in response to environmental stimuli, including risk factors for CHD such as smoking. Therefore, progress in the field of epigenetics may open a new world for understanding the disease pathology and evaluating the risk of CHD.

Epigenetics refers to the reversible, stable, and heritable modifications in gene activity or function that are not encoded on the nucleotide sequence (12). DNA methylation is a crucial epigenetic silencing mechanism (13), typically regulating tissue-specific gene expression, genomic imprinting, and X chromosome inactivation (14). Lund et al. have found that changes in peripheral blood mononuclear cell (PBMC) and aortic DNA methylation occur before the appearance of any histologically detectable vascular lesions in a mouse model, suggesting that abnormal changes in DNA methylation may be the early signs of atherosclerosis (15).

Previous studies have suggested that DNA methylation in the blood may play an important role in the development of CHD (16). A novel and reproducible association between blood-based methylation levels at 47 CpG sites and acute coronary syndrome (ACS) has been identified in the Chinese population. In another cohort, 34 CpG sites have been discovered to be associated with acute myocardial infarction (AMI) in European and American populations (17). To note, Agha et al. (18) have reported that the methylation levels of blood-derived DNA could predict the risk of future CHD across diverse populations, indicating DNA methylation would be a promising biomarker for CHD.

β-actin, encoded by *ACTB*, is an important cytoskeletal protein and plays an essential role in cell division, migration, invasion, vesicle trafficking, and cell structure regulation (19). Actin cytoskeleton could regulate endothelial nitric oxide synthase (eNOS) activity to alter the production of NO and subsequently lead to endothelial dysfunction, which is the underlying pathogenesis of CHD (20). Moreover, redox regulation of actin cytoskeletal signaling may influence vascular remodeling and dysfunction, contributing to the development of vascular diseases (21). Therefore, it is valuable to investigate the relationship between the methylation status of *ACTB* and vascular diseases. Our previous study has identified blood-based *ACTB* hypomethylation as early as 2 years before the clinical status of stroke in a prospective cohort study (22). CHD is another common disease related to blood vessels. So far, there is no report on the relationship between blood-based *ACTB* methylation and CHD. Here, we performed quantitative mass spectrometry to investigate the association between *ACTB* methylation in peripheral blood and CHD by a case-control study in the Chinese population, which may provide potential new biomarkers for the risk evaluation of CHD.

## Materials and methods

### Study population

This study was approved by the Ethics Committee of Chinese PLA General Hospital. The written informed consents have been obtained from all subjects involved in the study. In total, 281 CHD cases and 272 gender-matched controls were recruited from the Chinese PLA General Hospital from 2018 to 2019. The blood samples of all the participants were collected at their first registration in our study center. The CHD patients had a median age of 61 years (53.5–70.0 years old), and the controls had a median age of 57 years (51.0–64.0 years old). The CHD cases were confirmed by coronary angiography combined with analysis of clinical manifestations. Among them, 197 suffered from heart failure (HF), and 75 had experienced myocardial infarction (MI). The cardiac function of the CHD cases with HF was assessed according to the New York Heart Association (NYHA) classification scheme as NYHA I (*n* = 63) and NYHA II (*n* = 90), which represent the minor to medium cardiac function impairment, as well as NYHA III (*n* = 36) and NYHA IV (*n* = 8) (23). The controls were randomly selected from annual health examination programs. All the controls were self-claimed healthy, had normal blood counts and had no history of CHD, cancer, or autoimmune diseases. No further exclusion or inclusion criteria were applied for the recruitment of controls. The detailed clinical characteristics of CHD cases and controls are listed in Table 1.

**Table 1:** Baseline characteristics of coronary heart disease (CHD) patients and controls.

**Characteristics**	**Controls (*N* = 272)**	**CHD cases (*N* = 281)**	**χ^2^**	***p–*value^a^**
	***N* (%)**	***N* (%)**		
**Gender**				
Female	90(33.1)	101(35.9)		
Male	182(66.9)	180(64.1)	0.498	0.480
**Smoking**				
No	174(64.0)	159(56.6)		
Yes	89(32.7)	122(43.4)		
Unknown	9(3.3)	0(0.0)	5.247	**0.022**
**Drinking**				
No	148(54.4)	181(64.4)		
Yes	115(42.3)	100(35.6)		
Unknown	9(3.3)	0(0.0)	3.765	0.052
**Hypertension**				
No	151(55.5)	82(29.2)		
Yes	106(39.0)	199(70.8)		
Unknown	15(5.5)	0(0.0)	47.815	**4.68E−12**
**Diabetes**				
No	197(72.4)	191(68.0)		
Yes	59(21.7)	90(32.0)		
Unknown	16(5.9)	0(0.0)	5.390	**0.020**
**Characteristics**	**Controls** **(*****N*** = **272)**	**CHD cases (*****N*** = **281)**	**Z**	***p–*** **value** ^b^
	**Median (IQR)**	**Median (IQR)**		
Age	57(51–64)	61(53.5–70)	−3.403	**0.001**
Total cholesterol (TC) (mmol/L)	4.42(3.72–5.14)	3.91(3.28–4.60)	−4.978	**6.41E−07**
Triglyceride (TG) (mmol/L)	1.48(1.07–2.22)	1.30(0.98–1.98)	−2.227	**0.026**
High density lipoprotein cholesterol (HDL–C) (mmol/L)	1.13(0.92–1.36)	1.07(0.90–1.30)	−1.621	0.105
Low density lipoprotein cholesterol (LDL–C) (mmol/L)	2.88(2.17–3.46)	2.33(1.87–2.91)	−5.313	**1.08E−07**

IQR, interquartile range.

^a^The p–values were calculated by the Chi–square test, and significant p–values are in bold.

^b^The p–values were calculated by the Mann–Whitney test, and significant p–values are in bold.

### Sample collection and processing

The peripheral blood samples were collected in Ethylene Diamine Tetraacetic Acid (EDTA) tubes and stored at −80°C until usage. Genomic DNA was isolated from peripheral whole blood using DNA Extraction Kit (TANTICA, Nanjing, China) according to the manufacturer's instructions.

### Bisulfite conversion

The genomic DNA was subjected to bisulfite conversion with the EZ-96 DNA Methylation Gold kit (Zymo Research Corporation, Orange, CA, USA) according to the standard protocol. The bisulfite treatment converts non-methylated cytosine (C) at the CpG site to uracil (U), while methylated cytosine remains intact. The samples from CHD cases and controls were treated and analyzed in parallel throughout all the processes.

### MALDI-TOF mass spectrometry

**Figure 1:**
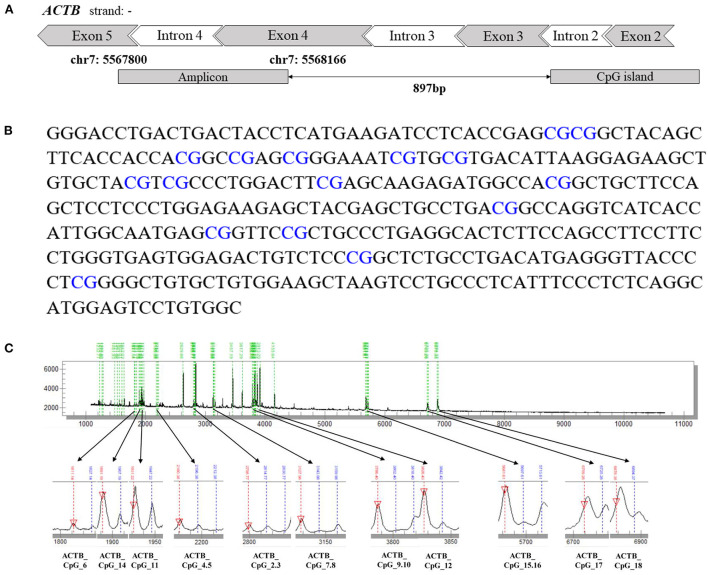
The schematic diagram, sequence and the mass peaks of *ACTB* amplicon. **(A)** The location of the investigated 367 bp amplicon in *ACTB* (from the UCSC Genome Browser). **(B)** The sequence of the *ACTB* amplicon examined by the EpiTyper assay (chr7: 5567800–5568166, build 37/hg 19, defined by the UCSC Genome Browser). The 16 measurable CpG sites are depicted in blue. **(C)** The MassARRAY assay yielded 11 distinguishable peaks. The downward red arrow pointing at the signal peak indicates the completely non-methylated CpG site, and the blue line above the signal peak indicates the methylated CpG site. The sample mass of the signal peak is represented at the bottom.

Agena MALDI-TOF (matrix-assisted laser desorption ionization time-of-flight) mass spectrometry (Agena Bioscience, San Diego, CA, USA) described by Yang et al. (24) and Yin et al. (25) was used to determine the level of DNA methylation quantitatively. Briefly, the bisulfite-converted DNA was amplified by bisulfite-specific primers (Supplementary Table 1). The PCR products were incubated with shrimp alkaline phosphatase (SAP) and further treated by T7 transcriptase along with RNase according to the manufacturer's instructions of Agena EpiTyper Assay. After being cleaned by resin, the final products were dispensed to a 384 SpectroCHIP by a Nanodispenser (Agena Bioscience, San Diego, CA, USA). The CHIP was read by a MassARRAY system (Agena Bioscience, San Diego, CA, USA). Data were obtained with SpectroACQUIRE v3.3.1.3 software (Agena Bioscience, San Diego, CA, USA) and visualized with EpiTyper v1.2 software (Agena Bioscience, San Diego, CA, USA). In this method, the conversion of unmethylated cytosine to uracil during bisulfite treatment generated base-specific cleavage products that reflected underlying methylation patterns. Subsequently, the status-specific mass signals were detected by MALDI-TOF mass spectrometry to determine which CpGs in the template sequence were methylated, and the ratio of the peak areas of corresponding mass signals was used to estimate the relative methylation level (26). The MassArray generated measurable data for 16 CpG sites in the amplicon of *ACTB* (367bp, chr7: 5567800-5568166) and yielded 11 distinguishable mass peaks. The schematic diagram and the sequence of *ACTB* amplicon are presented in Figures 1A,B. Neither the primers nor the CpG sites in the amplicon are overlapped with any known SNPs. Most of the mass peaks have only one CpG locus, and a few contain two. For example, ACTB_CpG_2.3 means ACTB_CpG_2 and ACTB_CpG_3 located at the same fragment after the EpiTyper treatment, and thus the methylation levels of ACTB_CpG_2 and ACTB_CpG_3 were presented as an average, which is also applied to ACTB_CpG_4.5, ACTB_CpG_7.8, ACTB_CpG_9.10, and ACTB_CpG_15.16. The samples from CHD cases and controls were treated and analyzed in parallel throughout all processes. The same numbers of cases and controls were analyzed on each chip for the analyses of MassARRAY.

### Statistical analysis

SPSS statistics 25.0 (IBM, NY, USA) and GraphPad Prism software version 8.0 (GraphPad Software, San Diego, California, USA) were used for statistical analyses of the data. Quantitative variables with non-Gaussian distribution, such as the levels of total cholesterol (TC), triglycerides (TG), high-density lipoprotein cholesterol (HDL-C), low-density lipoprotein cholesterol (LDL-C), and the methylation level of *ACTB* were expressed as median [interquartile range (IQR)], and the differences between cases and controls were assessed with Mann-Whitney U tests and Kruskal-Wallis tests. Qualitative variables were compared using the Chi-square (χ^2^) test. Odds ratios (ORs) and 95% confidence intervals (CIs) were estimated by logistic regression models adjusted for possible and available covariates, especially for the significant covariates as indicated in Table 1. The correlations were assessed by Spearman's rank correlation coefficients (r) and contingency coefficients (C). Besides, correlation analysis was conducted to discover the relationships among methylation levels at different CpG sites in the *ACTB* amplicon. Then, we used principal component analysis (PCA) to identify common underlying factors that better explain the largest variation in *ACTB* methylation. Criteria for factor selection were eigenvalue > 1.0 as revealed by the scree test. After that, each subject received a factor score, calculated by summing the observed methylation site values, each weighted by factor loadings (27–29). Receiver operating characteristic (ROC) curve analysis was applied to assess the discriminatory power of methylation levels. All statistical tests were two-sided, and *p*-values < 0.05 were defined as statistically significant.

## Results

### Hypermethylation of *ACTB* is associated with CHD

To evaluate the association between *ACTB* methylation in the peripheral blood and CHD, the same amplicon as described by Liu et al. (22) harboring 11 distinguished CpG groups (labeled as ACTB_CpG_2.3, ACTB_CpG_4.5, ACTB_CpG_6, ACTB_CpG_7.8, ACTB_CpG_9.10, ACTB_CpG_11, ACTB_CpG_12, ACTB_CpG_14, ACTB_CpG_15.16, ACTB_CpG_17, and ACTB_CpG_18) were amplified and analyzed by Agena MALDI-TOF mass spectrometry in 281 patients with CHD and 272 CHD-free controls. The complete map of mass spectrum with all the CpG sites tested in the *ACTB* amplicon was presented in Figure 1C. The reproducibility of mass spectrometry for the methylation analyses was evaluated in the present study. The tested DNA sample was randomly selected and measured by mass spectrometry for five times (Supplementary Table 2). ACTB_CpG_4.5 and ACTB_CpG_6 were excluded in the subsequent analysis due to low quality of signal and unsatisfying standard deviations (≥ 5%) (27). The association was analyzed by conducting two logistic regression models adjusted for different covariates (model 1: adjusted for age, gender, and batch effect, and model 2: adjusted for all the baseline characteristics in Table 1 that had significant differences between CHD cases and controls).

Nine CpG sites in the *ACTB* amplicon showed significantly higher methylation in CHD cases compared to controls (ACTB_CpG_2.3, ACTB_CpG_7.8, ACTB_CpG_9.10, ACTB_CpG_12, and ACTB_CpG_15.16, ORs per +10% methylation ranging from 1.19 to 1.45, *p* < 0.013, FDR-adjusted *p*-value < 0.019 for all by logistic regression model 2, Table 2). Apart from these, no significant associations were observed between other four *ACTB* CpG sites and CHD (*p* > 0.05 for all by logistic regression model 2, Table 2). In addition, the methylation levels of all CpG sites in the *ACTB* amplicon were positively correlated with each other, and the correlation was stronger if two CpG sites were closer (Supplementary Figure 1).

**Table 2:** Methylation difference of *ACTB* between CHD cases and controls.

**CpG sites**	**Controls** **(*N* = 272)**	**CHD cases** **(*N* = 281)**	**Model 1** ^**a**^		**Model 2** ^**b**^		**FDR**
	**Median (IQR)**	**Median (IQR)**	**OR (95%CI) per +10% methylation**	***p–*value**	**OR (95%CI) per +10% methylation**	***p–*value**	
ACTB_CpG_2.3	0.48(0.40–0.57)	0.54(0.45–0.65)	1.41(1.24–1.60)	**1.11E−07**	1.36(1.18–1.57)	**2.10E−05**	**1.37E−04**
ACTB_CpG_7.8	0.35(0.27–0.42)	0.40(0.30–0.50)	1.35(1.19–1.54)	**4.00E−06**	1.32(1.14–1.52)	**1.44E−04**	**3.12E−04**
ACTB_CpG_9.10	0.30(0.23–0.36)	0.36(0.28–0.42)	1.51(1.28–1.78)	**7.36E−07**	1.45(1.21–1.73)	**5.40E−05**	**1.76E−04**
ACTB_CpG_11	0.70(0.61–0.81)	0.70(0.59–0.80)	0.99(0.90–1.09)	0.820	0.96(0.86–1.07)	0.407	0.416
ACTB_CpG_12	0.25(0.17–0.33)	0.27(0.18–0.36)	1.20(1.06–1.36)	**0.004**	1.19(1.04–1.37)	**0.013**	**0.019**
ACTB_CpG_14	0.54(0.43–0.72)	0.53(0.39–0.69)	0.96(0.88–1.05)	0.355	0.94(0.85–1.03)	0.197	0.233
ACTB_CpG_15.16	0.53(0.42–0.62)	0.57(0.49–0.65)	1.25(1.11–1.41)	**3.33E−04**	1.19(1.04–1.36)	**0.012**	**0.019**
ACTB_CpG_17	0.33(0.26–0.40)	0.34(0.27–0.44)	1.11(0.99–1.24)	0.067	1.11(0.98–1.26)	0.112	0.146
ACTB_CpG_18	0.32(0.25–0.39)	0.33(0.26–0.41)	1.10(0.98–1.23)	0.096	1.05(0.93–1.19)	0.416	0.416

OR, odds ratio; CI, confidence interval; FDR, false discovery rate.

^a^Model 1: Logistic regression adjusted for age, gender, and batch.

^b^Model 2: Logistic regression adjusted for age, gender, smoking, hypertension, diabetes, TC, TG, LDL–C, and batch effect. Significant p–values are in bold.

Because methylation levels of *ACTB* CpG sites showed significant inter-correlations (Supplementary Figure 1), we then used PCA to identify common underlying factors that better explain the largest variation in *ACTB* methylation. Two main methylation factors finally emerged with PCA, explaining 54.03 % of gene methylation variance. Factor 1 was characterized by high positive loadings of ACTB_CpG_2.3, ACTB_CpG_15.16, ACTB_CpG_7.8, ACTB_CpG_17, ACTB_CpG_12, and ACTB_CpG_9.10, and Factor 2 showed high loadings of ACTB_CpG_14, ACTB_CpG_11, and ACTB_CpG_18 (Supplementary Table 3). We found a significant association between hypermethylation of Factor 1 and CHD [OR per +10% methylation (95% CI) = 1.66 (1.33–2.06), *p* = 7.00 × 10^−6^ by logistic regression model 2, Supplementary Table 4].

### The influence of gender and age on the methylation level of *ACTB*

Gender and age have been reported to play essential roles in the patterns of DNA methylation (30, 31). We therefore investigated the association between the methylation of *ACTB* and CHD stratified by gender and age, respectively. Interestingly, the CHD-associated *ACTB* methylation varied by gender. In females, only four CpG sites showed higher methylation levels in CHD cases than in controls [for ACTB_CpG_2.3, OR per +10% methylation (95% CI) = 1.63 (1.25–2.14), *p* = 3.99 × 10^−4^; for ACTB_CpG_9.10, OR per +10% methylation (95% CI) = 1.53 (1.12–2.09), *p* = 0.007, logistic regression model 2, Table 3A]. The association was enhanced in male group with nine CpG sites exhibiting significantly higher methylation levels in CHD cases (ACTB_CpG_2.3, ACTB_CpG_7.8, ACTB_CpG_9.10, ACTB_CpG_12, and ACTB_CpG_15.16, ORs per +10% methylation ranging from 1.20 to 1.43, *p* < 0.030 for all by logistic regression model 2, Table 3B). Among them, ACTB_CpG_7.8 showed the most significant difference [OR per +10% methylation (95% CI) = 1.38 (1.15–1.66), *p* = 4.81 × 10^−4^ by logistic regression model 2, Table 3B]. Factor 1 was significantly associated with CHD in male group [OR per +10% methylation (95% CI) = 1.63 (1.24–2.14), *p* = 4.42 × 10^−4^ by logistic regression model 2, Supplementary Table 5].

**Table 3:** Gender–specific methylation difference of *ACTB* between CHD cases and controls.

**CpG sites**	**Controls** **(*N* = 90)**	**CHD cases** **(*N* = 101)**	**Model 1** ^**a**^		**Model 2** ^**b**^	
	**Median (IQR)**	**Median (IQR)**	**OR (95%CI) per +10% methylation**	***p–*value**	**OR (95%CI) per +10% methylation**	***p–*value**
**A. Female**						
ACTB_CpG_2.3	0.45(0.38–0.53)	0.55(0.44–0.65)	1.58(1.26–1.98)	**9.20E−05**	1.63(1.25–2.14)	**3.99E−04**
ACTB_CpG_7.8	0.34(0.27–0.42)	0.37(0.29–0.45)	1.24(1.00–1.54)	**0.047**	1.24(0.98–1.57)	0.075
ACTB_CpG_9.10	0.29(0.23–0.35)	0.35(0.27–0.42)	1.54(1.17–2.03)	**0.002**	1.53(1.12–2.09)	**0.007**
ACTB_CpG_11	0.72(0.62–0.81)	0.70(0.60–0.80)	0.94(0.79–1.13)	0.515	0.89(0.73–1.08)	0.244
ACTB_CpG_12	0.26(0.17–0.35)	0.25(0.16–0.34)	1.09(0.88–1.36)	0.408	1.12(0.89–1.43)	0.336
ACTB_CpG_14	0.53(0.42–0.64)	0.51(0.41–0.64)	0.98(0.84–1.15)	0.820	0.97(0.81–1.15)	0.685
ACTB_CpG_15.16	0.50(0.41–0.62)	0.57(0.48–0.64)	1.26(1.02–1.55)	**0.031**	1.17(0.93–1.48)	0.191
ACTB_CpG_17	0.31(0.24–0.39)	0.33(0.27–0.44)	1.14(0.93–1.41)	0.200	1.11(0.88–1.41)	0.362
ACTB_CpG_18	0.29(0.22–0.36)	0.32(0.26–0.40)	1.28(0.99–1.65)	0.057	1.16(0.88–1.54)	0.304
**CpG sites**	**Controls** **(*****N*** = **182)**	**CHD cases** **(*****N*** = **180)**	**Model 1** ^a^		**Model 2** ^b^	
	**Median (IQR)**	**Median (IQR)**	**OR (95%CI) per** +**10% methylation**	***p–*** **value**	**OR (95%CI) per** +**10% methylation**	***p–*** **value**
**B. Male**						
ACTB_CpG_2.3	0.49(0.41–0.57)	0.54(0.46–0.66)	1.33(1.14–1.55)	**2.46E−04**	1.27(1.07–1.51)	**0.006**
ACTB_CpG_7.8	0.36(0.27–0.43)	0.41(0.32–0.52)	1.41(1.20–1.66)	**2.40E−05**	1.38(1.15–1.66)	**4.81E−04**
ACTB_CpG_9.10	0.31(0.24–0.37)	0.36(0.29–0.42)	1.49(1.22–1.83)	**1.17E−04**	1.43(1.14–1.79)	**0.002**
ACTB_CpG_11	0.68(0.60–0.80)	0.71(0.59–0.81)	1.00(0.89–1.13)	0.957	0.99(0.86–1.13)	0.834
ACTB_CpG_12	0.25(0.17–0.31)	0.28(0.20–0.37)	1.27(1.09–1.49)	**0.003**	1.26(1.05–1.50)	**0.013**
ACTB_CpG_14	0.55(0.43–0.75)	0.54(0.38–0.76)	0.94(0.85–1.05)	0.292	0.93(0.83–1.05)	0.247
ACTB_CpG_15.16	0.54(0.43–0.62)	0.58(0.49–0.67)	1.23(1.06–1.43)	**0.006**	1.20(1.02–1.42)	**0.030**
ACTB_CpG_17	0.34(0.27–0.41)	0.35(0.28–0.44)	1.09(0.95–1.25)	0.207	1.11(0.95–1.29)	0.185
ACTB_CpG_18	0.33(0.26–0.40)	0.34(0.26–0.42)	1.06(0.93–1.19)	0.402	1.03(0.90–1.19)	0.674

^a^Model 1: Logistic regression adjusted for age and batch.

^b^Model 2: Logistic regression adjusted for age, smoking, hypertension, diabetes, TC, TG, LDL–C, and batch effect. Significant p–values are in bold.

Furthermore, we stratified the subjects by the age of 60 years (the median age of subjects including cases and controls). In the group younger than 60 years old, seven CpG sites showed higher methylation levels in CHD cases than in controls (ACTB_CpG_2.3, ACTB_CpG_7.8, ACTB_CpG_9.10, and ACTB_CpG_12, ORs per +10% methylation ranging from 1.25 to 1.50, *p* < 0.026 for all by logistic regression model 2, Table 4A). In the group ≥ 60 years old, the methylation patterns of *ACTB* were similar as in the younger group, and eight CpG sites showed significantly higher methylation in CHD cases than in controls (ACTB_CpG_2.3, ACTB_CpG_7.8, ACTB_CpG_9.10, and ACTB_CpG_15.16, ORs per +10% methylation ranging from 1.33 to 1.71, *p* < 0.020 for all by logistic regression model 2, Table 4B). Among them, ACTB_CpG_2.3 showed the most significant difference [OR per +10% methylation (95% CI) = 1.71 (1.35–2.18), *p* = 1.10 × 10^−5^ by logistic regression model 2, Table 4B]. The association between hypermethylation of Factor 1 and CHD was similar in two age groups [for age < 60 years old, OR per +10% methylation (95% CI) = 1.70 (1.24–2.32), *p* = 0.001; for age ≥ 60 years old, OR per +10% methylation (95% CI) = 1.79 (1.27–2.51), *p* = 0.001, logistic regression model 2, Supplementary Table 6].

**Table 4:** Age–specific methylation difference of *ACTB* between CHD cases and controls.

**CpG sites**	**Controls** **(*N* = 143)**	**CHD cases** **(*N* = 125)**	**Model 1** ^**a**^		**Model 2** ^**b**^	
	**Median (IQR)**	**Median (IQR)**	**OR (95%CI) per +10% methylation**	***p–*value**	**OR (95%CI) per +10% methylation**	***p–*value**
**A. Age**<**60 years**						
ACTB_CpG_2.3	0.47(0.37–0.57)	0.54(0.46–0.68)	1.38(1.16–1.63)	**2.01E−04**	1.25(1.03–1.52)	**0.026**
ACTB_CpG_7.8	0.32(0.24–0.44)	0.40(0.30–0.53)	1.40(1.19–1.66)	**6.80E−05**	1.35(1.11–1.64)	**0.002**
ACTB_CpG_9.10	0.29(0.22–0.36)	0.36(0.29–0.43)	1.68(1.34–2.11)	**9.00E−06**	1.50(1.16–1.94)	**0.002**
ACTB_CpG_11	0.71(0.60–0.82)	0.72(0.60–0.86)	1.01(0.88–1.15)	0.911	0.96(0.83–1.12)	0.623
ACTB_CpG_12	0.21(0.13–0.32)	0.29(0.18–0.39)	1.34(1.14–1.58)	**0.001**	1.32(1.09–1.61)	**0.005**
ACTB_CpG_14	0.61(0.39–0.94)	0.61(0.39–0.89)	0.94(0.84–1.06)	0.315	0.90(0.79–1.03)	0.125
ACTB_CpG_15.16	0.53(0.40–0.63)	0.57(0.48–0.65)	1.18(1.02–1.37)	**0.031**	1.15(0.96–1.37)	0.121
ACTB_CpG_17	0.33(0.23–0.41)	0.35(0.28–0.46)	1.09(0.94–1.26)	0.241	1.11(0.94–1.32)	0.219
ACTB_CpG_18	0.30(0.21–0.41)	0.33(0.26–0.41)	1.05(0.92–1.21)	0.444	1.04(0.88–1.23)	0.630
**CpG sites**	**Controls** **(*****N*** = **129)**	**CHD cases** **(*****N*** = **156)**	**Model 1** ^a^		**Model 2** ^b^	
	**Median (IQR)**	**Median (IQR)**	**OR (95%CI) per** +**10% methylation**	***p–*** **value**	**OR (95%CI) per** +**10% methylation**	***p–*** **value**
**B. Age** ≥**60 years**						
ACTB_CpG_2.3	0.48(0.42–0.56)	0.54(0.45–0.64)	1.53(1.25–1.87)	**3.40E−05**	1.71(1.35–2.18)	**1.10E−05**
ACTB_CpG_7.8	0.36(0.30–0.42)	0.39(0.30–0.47)	1.33(1.08–1.64)	**0.008**	1.33(1.06–1.66)	**0.015**
ACTB_CpG_9.10	0.31(0.26–0.37)	0.35(0.27–0.42)	1.34(1.05–1.70)	**0.017**	1.36(1.05–1.77)	**0.020**
ACTB_CpG_11	0.68(0.61–0.79)	0.70(0.58–0.77)	0.97(0.83–1.14)	0.695	0.92(0.77–1.09)	0.334
ACTB_CpG_12	0.28(0.23–0.34)	0.25(0.19–0.34)	1.04(0.85–1.28)	0.710	1.06(0.85–1.33)	0.585
ACTB_CpG_14	0.52(0.46–0.59)	0.50(0.39–0.60)	0.96(0.83–1.11)	0.571	0.94(0.80–1.10)	0.403
ACTB_CpG_15.16	0.53(0.45–0.61)	0.57(0.50–0.66)	1.41(1.15–1.73)	**0.001**	1.33(1.06–1.67)	**0.013**
ACTB_CpG_17	0.34(0.28–0.40)	0.33(0.27–0.43)	1.17(0.97–1.40)	0.096	1.17(0.96–1.43)	0.116
ACTB_CpG_18	0.32(0.28–0.38)	0.34(0.26–0.41)	1.21(1.00–1.48)	0.056	1.14(0.92–1.42)	0.229

^a^Model 1: Logistic regression adjusted for age, gender, and batch.

^b^Model 2: Logistic regression adjusted for age, gender, smoking, hypertension, diabetes, TC, TG, LDL–C, and batch effect. Significant p–values are in bold.

### Methylation difference of *ACTB* between patients with MI, HF, and controls

Of the 281 CHD patients, 75 had experienced MI and 197 suffered from HF. We further investigated the association between these CHD subtypes and the blood-based methylation level of *ACTB*. The association between *ACTB* hypermethylation and CHD was mainly originated from the non-MI CHD cases, where nine CpG sites exhibited significantly higher methylation levels in the non-MI CHD cases than in the controls (ACTB_CpG_2.3, ACTB_CpG_7.8, ACTB_CpG_9.10, ACTB_CpG_15.16, and ACTB_CpG_17, ORs per +10% methylation ranging from 1.16 to 1.53, *p* < 0.035 for all by logistic regression model 2, Table 5A). Among them, the most significant locus was ACTB_CpG_2.3 with an OR of 1.52 per +10% methylation and a *p*-value of 6.72 × 10^−7^ (logistic regression model 2, Table 5A). Only three CpG sites showed increased methylation in the MI cases [for ACTB_CpG_7.8, OR per +10% methylation (95% CI) = 1.40 (1.12–1.75), *p* = 0.003; for ACTB_CpG_12, OR per +10% methylation (95% CI) = 1.33 (1.06–1.67), *p* = 0.012; logistic regression model 2, Table 5B]. Unexpectedly, the methylation at ACTB_CpG_14 was significantly decreased in the MI cases comparing to the controls [for ACTB_CpG_14, median = 0.43 (IQR = 0.28–0.58) and 0.54 (IQR = 0.43–0.72) for MI CHD cases and controls, respectively, OR per +10% methylation (95% CI) = 0.70 (0.59–0.83), *p* = 4.40 × 10^−5^ by logistic regression model 2, Table 5B]. Factor 1 showed increased methylation in the non-MI CHD cases while Factor 2 showed decreased methylation in the MI CHD cases [for Factor 1, OR per +10% methylation (95% CI) = 1.64 (1.28–2.09), *p* = 7.50 × 10^−5^; for Factor 2, OR per +10% methylation (95% CI) = 0.43 (0.29–0.64), *p* = 4.20 × 10^−5^, logistic regression model 2, Supplementary Table 7].

**Table 5:** Methylation difference of *ACTB* between non–MI CHD cases, MI CHD cases and controls.

**CpG sites**	**Controls** **(*N* = 272)**	**Non–MI CHD cases** **(*N* = 206)**	**Model 1** ^**a**^		**Model 2** ^**b**^	
	**Median (IQR)**	**Median (IQR)**	**OR (95%CI) per +10% methylation**	***p–*value**	**OR (95%CI) per +10% methylation**	***p–*value**
**A. Non–MI CHD cases vs. controls**						
ACTB_CpG_2.3	0.48(0.40–0.57)	0.56(0.47–0.67)	1.56(1.35–1.80)	**2.96E−09**	1.52(1.29–1.80)	**6.72E−07**
ACTB_CpG_7.8	0.35(0.27–0.42)	0.39(0.30–0.50)	1.35(1.17–1.56)	**3.40E−05**	1.30(1.11–1.53)	**0.001**
ACTB_CpG_9.10	0.30(0.23–0.36)	0.36(0.28–0.42)	1.63(1.35–1.97)	**2.71E−07**	1.53(1.25–1.88)	**5.10E−05**
ACTB_CpG_11	0.70(0.61–0.81)	0.71(0.61–0.82)	1.05(0.94–1.17)	0.377	0.99(0.88–1.12)	0.894
ACTB_CpG_12	0.25(0.17–0.33)	0.26(0.17–0.35)	1.16(1.01–1.33)	**0.033**	1.16(0.99–1.35)	0.065
ACTB_CpG_14	0.54(0.43–0.72)	0.56(0.42–0.82)	1.06(0.96–1.17)	**2.46E−01**	1.04(0.93–1.17)	0.459
ACTB_CpG_15.16	0.53(0.42–0.62)	0.57(0.49–0.67)	1.27(1.12–1.45)	**3.32E−04**	1.20(1.04–1.39)	**0.014**
ACTB_CpG_17	0.33(0.26–0.40)	0.35(0.28–0.46)	1.16(1.03–1.31)	**0.015**	1.16(1.01–1.33)	**0.035**
ACTB_CpG_18	0.32(0.25–0.39)	0.34(0.26–0.42)	1.16(1.02–1.31)	**0.021**	1.13(0.98–1.30)	0.093
**CpG sites**	**Controls** **(*****N*** = **272)**	**MI CHD cases** **(*****N*** = **75)**	**Model 1** ^a^		**Model 2** ^b^	
	**Median (IQR)**	**Median (IQR)**	**OR (95%CI) per** +**10% methylation**	***p–*** **value**	**OR (95%CI) per** +**10% methylation**	***p–*** **value**
**B. MI CHD cases vs. controls**						
ACTB_CpG_2.3	0.48(0.40–0.57)	0.51(0.39–0.59)	1.13(0.93–1.37)	0.237	1.11(0.91–1.37)	0.309
ACTB_CpG_7.8	0.35(0.27–0.42)	0.40(0.30–0.49)	1.42(1.15–1.74)	**0.001**	1.40(1.12–1.75)	**0.003**
ACTB_CpG_9.10	0.30(0.23–0.36)	0.34(0.27–0.42)	1.27(0.99–1.62)	0.061	1.23(0.96–1.59)	0.107
ACTB_CpG_11	0.70(0.61–0.81)	0.68(0.52–0.77)	0.85(0.72–1.00)	**0.048**	0.85(0.72–1.01)	0.070
ACTB_CpG_12	0.25(0.17–0.33)	0.28(0.21–0.37)	1.35(1.09–1.66)	**0.006**	1.33(1.06–1.67)	**0.012**
ACTB_CpG_14	0.54(0.43–0.72)	0.43(0.28–0.58)	0.73(0.62–0.85)	**5.40E−05**	0.70(0.59–0.83)	**4.40E−05**
ACTB_CpG_15.16	0.53(0.42–0.62)	0.58(0.48–0.65)	1.20(0.98–1.45)	0.072	1.18(0.96–1.45)	0.118
ACTB_CpG_17	0.33(0.26–0.40)	0.33(0.26–0.41)	0.93(0.75–1.14)	0.472	0.97(0.78–1.21)	0.779
ACTB_CpG_18	0.32(0.25–0.39)	0.31(0.24–0.39)	0.98(0.81–1.20)	0.858	0.94(0.76–1.16)	0.566

MI, myocardial infarction.

^a^Model 1: Logistic regression adjusted for age, gender, and batch.

^b^Model 2: Logistic regression adjusted for age, gender, smoking, hypertension, diabetes, TC, TG, LDL–C, and batch effect. Significant p–values are in bold.

Next, we assessed the association between the methylation level of *ACTB* and the status of HF. The methylation levels of six CpG sites in the *ACTB* amplicon were higher in HF cases than that in controls [for ACTB_CpG_2.3, OR per +10% methylation (95% CI) = 1.39 (1.19–1.63), *p* = 3.10 × 10^−5^; for ACTB_CpG_7.8, OR per +10% methylation (95% CI) = 1.38 (1.18–1.62), *p* = 6.00 × 10^−5^; for ACTB_CpG_9.10, OR per +10% methylation (95% CI) = 1.46 (1.20–1.78), *p* = 1.52 × 10^−4^, logistic regression model 2, Table 6]. None of the other CpG loci in the *ACTB* amplicon showed an association with HF (*p* > 0.05 for all by logistic regression model 2, Table 6). In addition, hypermethylation of seven CpG sites showed a borderline association with non-HF CHD cases (ACTB_CpG_2.3, ACTB_CpG_9.10, ACTB_CpG_12, and ACTB_CpG_15.16, ORs per +10% methylation ranging from 1.28 to 1.33, *p* < 0.034 for all by logistic regression model 2, Supplementary Table 8). According to the guideline issued by AHA/ACC/HFSA in 2022 (32), the status of HF can be classified by left ventricular ejection fraction (LVEF). Among the 197 HF cases, 26 patients had HFrEF (HF with reduced EF, LVEF ≤ 40%), 15 patients had HFmrEF (HF with mildly reduced EF, LVEF 41% ~ 49%), and 156 patients had HFpEF (HF with preserved EF, LVEF ≥ 50%). The methylation levels of nine CpG sites were different between three types of HF cases and controls (ACTB_CpG_2.3, ACTB_CpG_7.8, ACTB_CpG_9.10, ACTB_CpG_14, and ACTB_CpG_15.16, *p* < 0.044 for all by Kruskal-Wallis test, Supplementary Table 9). Besides, the methylation level of Factor 1 was higher in HF cases than that in controls [OR per +10% methylation (95% CI) = 1.69 (1.33–2.14), *p* = 1.50 × 10^−5^ by logistic regression model 2, Supplementary Table 10].

**Table 6:** Methylation difference of *ACTB* between HF cases and controls.

**CpG sites**	**Controls** **(*N* = 272)**	**HF cases** **(*N* = 197)**	**Model 1** ^**a**^		**Model 2** ^**b**^	
	**Median (IQR)**	**Median (IQR)**	**OR (95%CI) per +10% methylation**	***p–*value**	**OR (95%CI) per +10% methylation**	***p–*value**
ACTB_CpG_2.3	0.48(0.40–0.57)	0.54(0.46–0.63)	1.42(1.23–1.64)	**2.00E−06**	1.39(1.19–1.63)	**3.10E−05**
ACTB_CpG_7.8	0.35(0.27–0.42)	0.40(0.32–0.50)	1.38(1.19–1.60)	**1.40E−05**	1.38(1.18–1.62)	**6.00E−05**
ACTB_CpG_9.10	0.30(0.23–0.36)	0.36(0.29–0.42)	1.52(1.26–1.83)	**9.00E−06**	1.46(1.20–1.78)	**1.52E−04**
ACTB_CpG_11	0.70(0.61–0.81)	0.70(0.58–0.80)	0.99(0.89–1.10)	0.826	0.96(0.85–1.08)	0.504
ACTB_CpG_12	0.25(0.17–0.33)	0.25(0.17–0.34)	1.16(1.01–1.33)	**0.036**	1.15(0.99–1.34)	0.072
ACTB_CpG_14	0.54(0.43–0.72)	0.49(0.37–0.62)	0.94(0.85–1.04)	0.225	0.92(0.82–1.02)	0.113
ACTB_CpG_15.16	0.53(0.42–0.62)	0.56(0.48–0.64)	1.19(1.04–1.36)	**0.009**	1.15(1.00–1.33)	0.052
ACTB_CpG_17	0.33(0.26–0.40)	0.33(0.27–0.42)	1.10(0.97–1.25)	0.121	1.11(0.97–1.27)	0.146
ACTB_CpG_18	0.32(0.25–0.39)	0.33(0.26–0.40)	1.15(1.01–1.30)	**0.031**	1.10(0.96–1.27)	0.174

HF, heart failure.

^a^Model 1: Logistic regression adjusted for age, gender, and batch.

^b^Model 2: Logistic regression adjusted for age, gender, smoking, hypertension, diabetes, TC, TG, LDL–C, and batch effect. Significant p–values are in bold.

### Methylation difference of *ACTB* between NYHA I and II CHD cases and controls

In our study, the cardiac function of 153 CHD cases was classified as NYHA I and NYHA II (NYHA I CHD cases = 63, NYHA II CHD cases = 90). Compared to the healthy controls, the methylation levels of ACTB_CpG_2.3, ACTB_CpG_7.8, and ACTB_CpG_9.10 were significantly increased in NYHA I&II cases [for ACTB_CpG_2.3, OR per +10% methylation (95% CI) = 1.39 (1.17–1.65), *p* = 1.91 × 10^−4^; for ACTB_CpG_7.8, OR per +10% methylation (95% CI) = 1.38 (1.14–1.66), *p* = 0.001; for ACTB_CpG_9.10, OR per +10% methylation (95% CI) = 1.44 (1.15–1.79), *p* = 0.001, logistic regression model 2, Table 7]. No significant association was observed for the other CpG sites (*p* > 0.05 for all by logistic regression model 2, Table 7). The methylation level of Factor 1 was significantly increased in NYHA I&II cases [OR per +10% methylation (95% CI) = 1.67 (1.28–2.18), *p* = 1.37 × 10^−4^ by logistic regression model 2, Supplementary Table 11].

**Table 7:** Methylation difference of *ACTB* between NYHA I and II CHD cases and controls.

**CpG sites**	**Controls** **(*N* = 272)**	**NYHA I&II CHD cases** **(*N* = 153)**	**Model 1** ^**a**^		**Model 2** ^**b**^	
	**Median (IQR)**	**Median (IQR)**	**OR (95%CI) per +10% methylation**	***p–*value**	**OR (95%CI) per +10% methylation**	***p–*value**
ACTB_CpG_2.3	0.48(0.40–0.57)	0.53(0.47–0.63)	1.45(1.24–1.69)	**4.00E−06**	1.39(1.17–1.65)	**1.91E−04**
ACTB_CpG_7.8	0.35(0.27–0.42)	0.40(0.33–0.49)	1.40(1.18–1.65)	**8.90E−05**	1.38(1.14–1.66)	**0.001**
ACTB_CpG_9.10	0.30(0.23–0.36)	0.36(0.29–0.42)	1.52(1.24–1.87)	**5.60E−05**	1.44(1.15–1.79)	**0.001**
ACTB_CpG_11	0.70(0.61–0.81)	0.71(0.59–0.80)	1.00(0.89–1.13)	0.974	0.96(0.84–1.09)	0.524
ACTB_CpG_12	0.25(0.17–0.33)	0.26(0.18–0.35)	1.21(1.03–1.41)	**0.018**	1.17(0.99–1.40)	0.071
ACTB_CpG_14	0.54(0.43–0.72)	0.50(0.37–0.62)	0.95(0.85–1.07)	0.407	0.92(0.81–1.04)	0.198
ACTB_CpG_15.16	0.53(0.42–0.62)	0.57(0.47–0.64)	1.20(1.04–1.38)	**0.015**	1.13(0.97–1.32)	0.132
ACTB_CpG_17	0.33(0.26–0.40)	0.33(0.26–0.42)	1.11(0.97–1.27)	0.143	1.09(0.94–1.27)	0.258
ACTB_CpG_18	0.32(0.25–0.39)	0.34(0.26–0.40)	1.22(1.06–1.41)	**0.006**	1.16(0.98–1.36)	0.082

NYHA, New York Heart Association.

^a^Model 1: Logistic regression adjusted for age, gender, and batch.

^b^Model 2: Logistic regression adjusted for age, gender, smoking, hypertension, diabetes, TC, TG, LDL–C, and batch effect. Significant p–values are in bold.

### The correlation between blood–based *ACTB* methylation and the clinical characteristics of CHD

Next, the relationship between *ACTB* methylation and the clinical characteristics (including smoking, alcohol drinking, hypertension, diabetes, and blood lipid levels) of 272 controls and 281 CHD cases was investigated. The methylation levels of all the CpG sites in the *ACTB* amplicon were inversely correlated with the level of HDL–C, but more significant in the CHD cases than in the controls (Supplementary Table 12). There was no or weak correlation between *ACTB* methylation and the other blood lipid indexes (levels of LDL–C, TC, TG), as well as the status of smoking, drinking, hypertension, and diabetes (Supplementary Table 12).

Most CHD patients had a history of taking medications, we therefore investigated the correlation between blood–based *ACTB* methylation and various medications. The use of β blocker, digoxin, aspirin, and statin was correlated with hypermethylation of *ACTB* (*p* < 0.047 for the four drugs, Supplementary Table 13). The other eight common cardiovascular drugs showed no obvious correlation with the methylation intensities of *ACTB* (Supplementary Table 13).

### *ACTB* methylation as a potential biomarker for the detection of CHD

**Figure 2:**
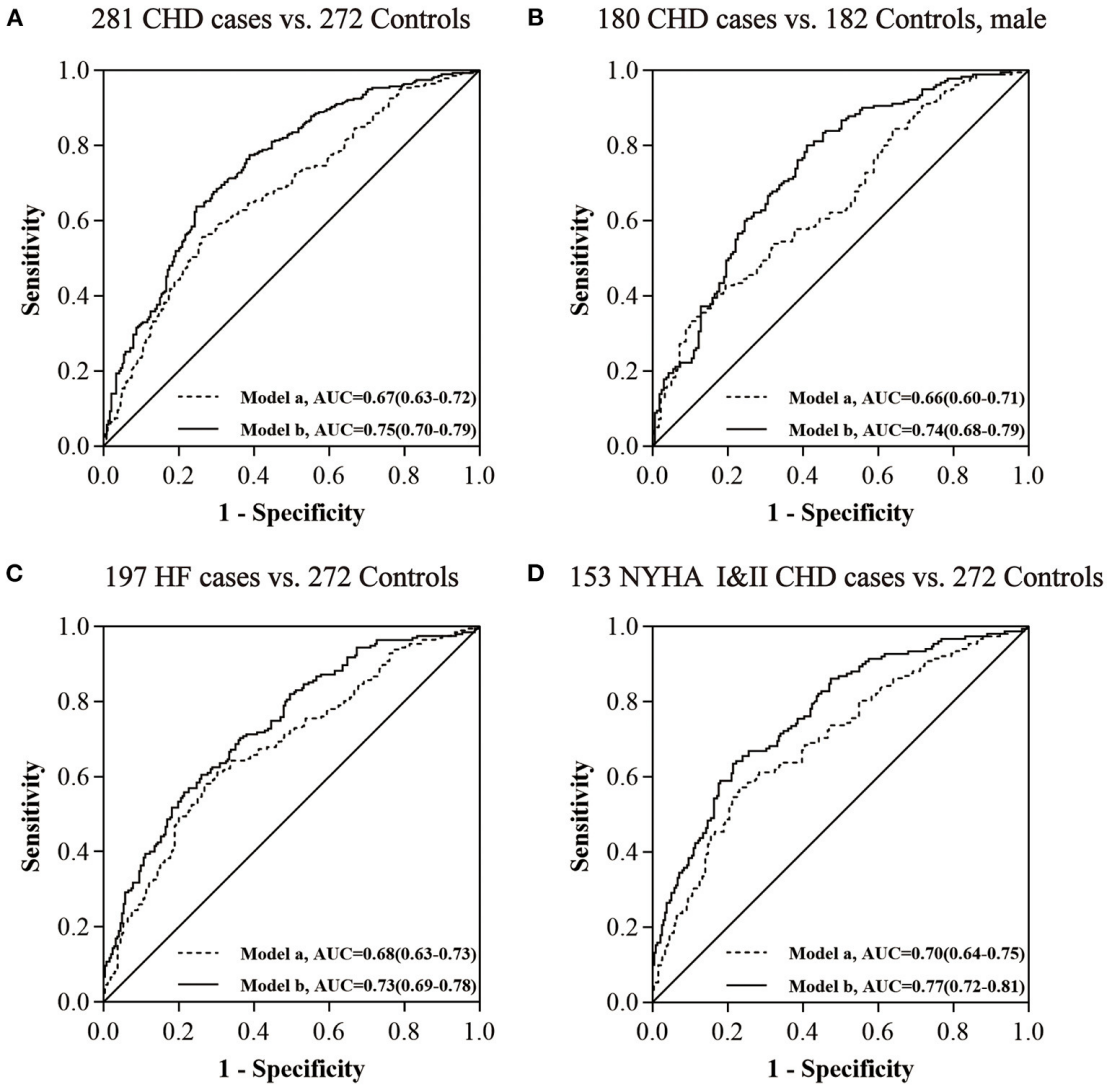
The power of *ACTB* (ACTB_CpG_2.3, ACTB_CpG_7.8, and ACTB_CpG_9.10) methylation to distinguish CHD cases from controls. **(A)** The discrimination of CHD cases from controls by *ACTB* methylation. **(B)** The discrimination of CHD cases from controls in males by *ACTB* methylation. **(C)** The discrimination of HF cases from controls by *ACTB* methylation. **(D)** The discrimination of NYHA I&II CHD cases from controls by *ACTB* methylation. The ROC analyses were calculated by logistic regression models (model a adjusted for age, gender, and batch effect, and model b adjusted for age, gender, smoking, hypertension, diabetes, TC, TG, LDL–C, and batch effect).

To estimate the potential clinical utility of *ACTB* methylation as a marker for the risk assessment of CHD, ROC curve analyses were performed adjusted for possible confounding effects by two logistic regression models (model a: adjusted for age, gender, and batch effect, and model b: adjusted for age, gender, smoking, hypertension, diabetes, TC, TG, LDL–C, and batch effect). The methylation levels of six CpG sites (ACTB_CpG_2.3, ACTB_CpG_7.8, and ACTB_CpG_9.10) in the *ACTB* amplicon exhibited efficient discriminatory power to distinguish general CHD cases, male CHD cases, HF cases, and NYHA I&II CHD cases from controls [area under curve (AUC) = 0.75, 0.74, 0.73, and 0.77, respectively, logistic regression model b; Figure 2, Supplementary Table 14]. When all measurable *ACTB* CpG sites were considered, the methylation level of *ACTB* showed a moderate discriminatory power (AUC = 0.75, 0.74, 0.74, and 0.76, respectively, logistic regression model b; Supplementary Table 14). Thus, compared to the six CpG sites (ACTB_CpG_2.3, ACTB_CpG_7.8, and ACTB_CpG_9.10), the discriminatory power could hardly be improved when all CpG sites in the *ACTB* amplicon were included in the models (Supplementary Table 14).

## Discussion

In the present study, we reported a strong association between hypermethylation of *ACTB* in peripheral blood and CHD. More specifically, the aberrant *ACTB* methylation is mainly correlated with CHD in the male group and HF status, and could even be detected in patients with minor to medium cardiac function impairment (NYHA I&II CHD cases).

β-actin (encoded by *ACTB*) is an important member in the actin family, and functions as a cytoskeleton protein that forms filaments. Actin cytoskeleton can regulate the activity of eNOS, which is related to the endothelial dysfunction. Recently, some researchers have suggested a link between CMD (coronary microvascular dysfunction), through endothelial dysfunction, and severity of symptoms in HF patients (33). For example, Borlaug et al. (34) assessed the microvasculature and found that the endothelial function was impaired in HFpEF patients compared with healthy controls, indicating the essential role of endothelial dysfunction in the development of HFpEF. Additionally, CMD is an important pathophysiological mechanism involved in myocardial infarction with non–obstructive coronary arteries (MINOCA), a syndrome generally observed in relatively young patients with lower prevalence of traditional cardiovascular risk factors (35, 36). And the microvascular dysfunction may trigger the myocardial ischemia in the absence of coronary obstructive disease (37). Therefore, it would be interesting to explore the underlying mechanisms of the relationship between altered methylation of *ACTB* and endothelial dysfunction and the consequent contribution to CVDs. Previous studies have found that β-actin plays a critical role in a wide range of cellular processes, including cell locomotion, migration, adhesion, division, intracellular transport, muscle contractility, and the regulation of gene expression (38–40). As a traditional housekeeping gene, *ACTB* is commonly used to normalize gene and protein expression in biological studies. However, the expression of *ACTB* may change in response to biochemical stimuli during cell growth and differentiation, and in some disease states (41, 42). Wang et al. (43) found that the expression of *ACTB* was significantly increased in epicardial adipose tissue of coronary artery disease (CAD) samples, which may contribute to the pathogenesis of CAD. Moreover, β-actin has been found to be a downstream effector of the RhoA and Rho kinase (ROCK) pathway, which regulates cell morphology by controlling cytoskeletal architecture and may regulate the vasoconstriction and vascular remodeling (44, 45). Karakozova et al. (46) reported that β-actin was arginylated *in vivo* to regulate actin filament properties, β-actin localization, and lamella formation in motile cells. Tondeleir et al. (47) revealed that the genetic down–regulation of *ACTB* could inhibit the migration of mouse embryonic fibroblasts. Thus, it is important to evaluate the regulatory effect of *ACTB* DNA methylation on gene expression. However, luciferase reporter gene assay may not be suitable for our study, because here we used MALDI–TOF mass spectrometry to quantitatively measure the methylation levels of CpG sites in the *ACTB* amplicon, but not just their methylated or unmethylated status. The mRNA materials would be helpful to provide additional evidence for the biological function of the methylation changes. Unfortunately, fresh blood samples were not available in our study populations, so that it was not possible to extract RNA for gene expression analysis. Whether the altered methylation of *ACTB* could modulate the gene expression and biological function requires further investigations in the future.

Yang et al. (48) reported that the expression of *ACTB* was significantly down–regulated in ischemic stroke cases who had a drinking habit. Our previous prospective study revealed the association between blood–based hypomethylation at ACTB_CpG_14 site and preclinical stroke patients, who mainly develop ischemic stroke 2 years after the baseline (22). Here, we used the same amplicon of *ACTB*, and to our surprise, decreased methylation level of ACTB_CpG_14 was only observed in MI CHD cases. MI and ischemic stroke are characterized by the build–up of plaque which decreases blood flow and eventually blocks the blood vessels (49, 50). The similar pathological foundation may explain why our study only revealed hypomethylation of ACTB_CpG_14 in MI. Nevertheless, the association between hypomethylation of ACTB_CpG_14 and the status of MI may be influenced by the limited sample size, and thus, validation in multi–center studies with enlarged sample sizes is necessary. On the other hand, blood–derived hypermethylation of *ACTB* was mainly associated with HF cases and could even be detected in the peripheral blood of patients with minor to medium cardiac function impairment (NYHA I&II CHD cases). These specific patterns of *ACTB* methylation broaden our horizon of the relationship between DNA methylation and CHD subtypes. Though limited by the sample size and statistical analysis, we noticed that the methylation levels of ACTB_CpG_2.3, ACTB_CpG_7.8 and ACTB_CpG_9.10 were increased in the HFrEF cases comparing to the controls, indicating that the aberrant DNA methylation pattern may play a role in the progress of HF.

Previous studies have revealed that the incidence of CHD is higher in males than in females (1, 51). We found the CHD–associated *ACTB* methylation varied by gender. In the males, nine of thirteen CpG loci in *ACTB* amplicon were related with CHD, including ACTB_CpG_2.3, ACTB_CpG_7.8, ACTB_CpG_9.10, ACTB_CpG_12, and ACTB_CpG_15.16. While in the females, only the methylation levels of ACTB_CpG_2.3 and ACTB_CpG_9.10 were associated with CHD. The different association patterns between males and females might be influenced by sexual hormones or other physical differences in gender and gender–related lifestyles (52). Therefore, it is reasonable to adjust the effect of gender when evaluating the association between DNA methylation and diseases. The males had higher *ACTB* methylation level than the females, and hypermethylation of *ACTB* is associated with increased risk for CHD according to our results. The gender–specific difference in *ACTB* methylation may provide further mechanistic support for the previous findings of a greater risk of CHD in males. Sex hormones may play an important role in determining DNA methylation levels, as well as exert regulatory effects on DNA methylation *via* downregulation of DNA methyltransferases expression in certain tissues (53, 54). The mean age of menopause in Asia is 48.8 years (55). There are only two female subjects lower than 50 years old in our study, indicating that most participants are in a post–menopausal condition. To explore the impact of sex hormones on the CHD associated hypermethylation of *ACTB*, future studies with larger sample size including information of menopausal status among female subjects and the presence of hormone replacement therapies would be helpful. There is an increased risk of CVD in older adults (56). However, the methylation patterns of *ACTB* were similar in two age groups in the present study, which suggested that CHD–associated *ACTB* methylation might be independent from age.

Our study analyzed the DNA methylation in whole blood and the methylation pattern can be affected by cell composition. Algorithms could be applied to adjusted for the cell populations in the unfractionated whole blood in the array–based methylation studies, which contains the information of cell component specific methylation signatures (57–60). However, such algorithms were not suitable for our candidate–gene approached study. White blood cell count could be a sufficient solution, but such information was not available in our study. In the ongoing study, we are collecting the white blood cell counts for each sample, and aiming to address the influence of cell population on the blood–based DNA methylation signatures.

Besides, the signatures of methylation could be influenced by environmental factors and treatment (61–67). Our results indicated that blood–based *ACTB* methylation was inversely correlated with the level of HDL–C, but not the levels of LDL–C, TC, and TG, or the status of smoking, drinking, hypertension, and diabetes. Moreover, we observed that the intake of β blocker, digoxin, aspirin, and statin was correlated with the hypermethylation of *ACTB* in the blood of CHD patients. Unexpectedly, the other eight common cardiovascular drugs showed no correlation with the blood–derived methylation level of *ACTB*. In addition, the medication histories of controls were not available in our present study. Since β blocker, aspirin, and statin are medicines widely prescribed on an outpatient basis and there is the possible presence of these therapies in controls, it is valuable to clarify the possible impact in terms of diagnostics by expanding the samples with future studies. The influence of CHD–related clinical factors and medication on the *ACTB* methylation needs further investigation with larger sample sizes and more detailed information. Nevertheless, our findings suggested that the methylation patterns in the blood may not be sensitive to most cardiovascular medications, and thus, the methylation signatures of *ACTB* might have the potential to be a biomarker for the risk evaluation or early detection of CHD in the future but not for the monitoring of treatment.

## Conclusion

In summary, this is the first study to report the association between hypermethylation of *ACTB* in peripheral blood and CHD in the Chinese population, especially in males and HF cases. Remarkably, hypermethylation of *ACTB* also existed in CHD patients with minor to medium cardiac function impairment. Moreover, this association is not or only weakly correlated with most of the environmental factors and common medical treatments. However, limited by retrospective sample, whether DNA methylation level in *ACTB* has a causal effect on CHD is unclear. Moreover, the use of cardiovascular medications may influence DNA methylation patterns of CHD patients. Prospective study bases on large sample size and follow–up would be helpful to address these points.

## Data availability statement

The original contributions presented in the study are included in the article/Supplementary material, further inquiries can be directed to the corresponding authors.

## Ethics statement

The studies involving human participants were reviewed and approved by the Ethics Committee of Chinese PLA General Hospital. The patients/participants provided their written informed consent to participate in this study.

## Author contributions

RY and XZ contributed to the conception and design of the study, critical revision of important intellectual content in the manuscript. JJ and CZ were responsible for performing the experiments and drafting the manuscript. JJ and RY were responsible for analyzing the results. JW and XZ provided the materials and supervised the patient enrollment and acquisition of biological samples and clinical data. All authors contributed to the article and approved the submitted version.

## Funding

This work was supported by the Nanjing Medical University Research Support Funding (grant number: 2018RC0003), National Natural Science Foundation of China (grant numbers: 82001994 and 82000311), Chinese PLA General Hospital Clinical Research Support Funding (grant number: 2018FC–WJFWZX−1–21), and Chinese PLA General Hospital Youth Development Project (grant number: QNC19058).

## Conflict of interest

The authors declare that the research was conducted in the absence of any commercial or financial relationships that could be construed as a potential conflict of interest.

## Publisher's note

All claims expressed in this article are solely those of the authors and do not necessarily represent those of their affiliated organizations, or those of the publisher, the editors and the reviewers. Any product that may be evaluated in this article, or claim that may be made by its manufacturer, is not guaranteed or endorsed by the publisher.
