# Supplementary material for: The association between ACTB methylation in peripheral blood and coronary heart disease in a case-control study
Source: Front Cardiovasc Med. 2022 Aug 18;9:972566. doi: 10.3389/fcvm.2022.972566 (PMC9433772; doi:10.3389/fcvm.2022.972566)
Supplement: Supplementary file 1 [file Data_Sheet_1.docx]

**Supplementary Figure**


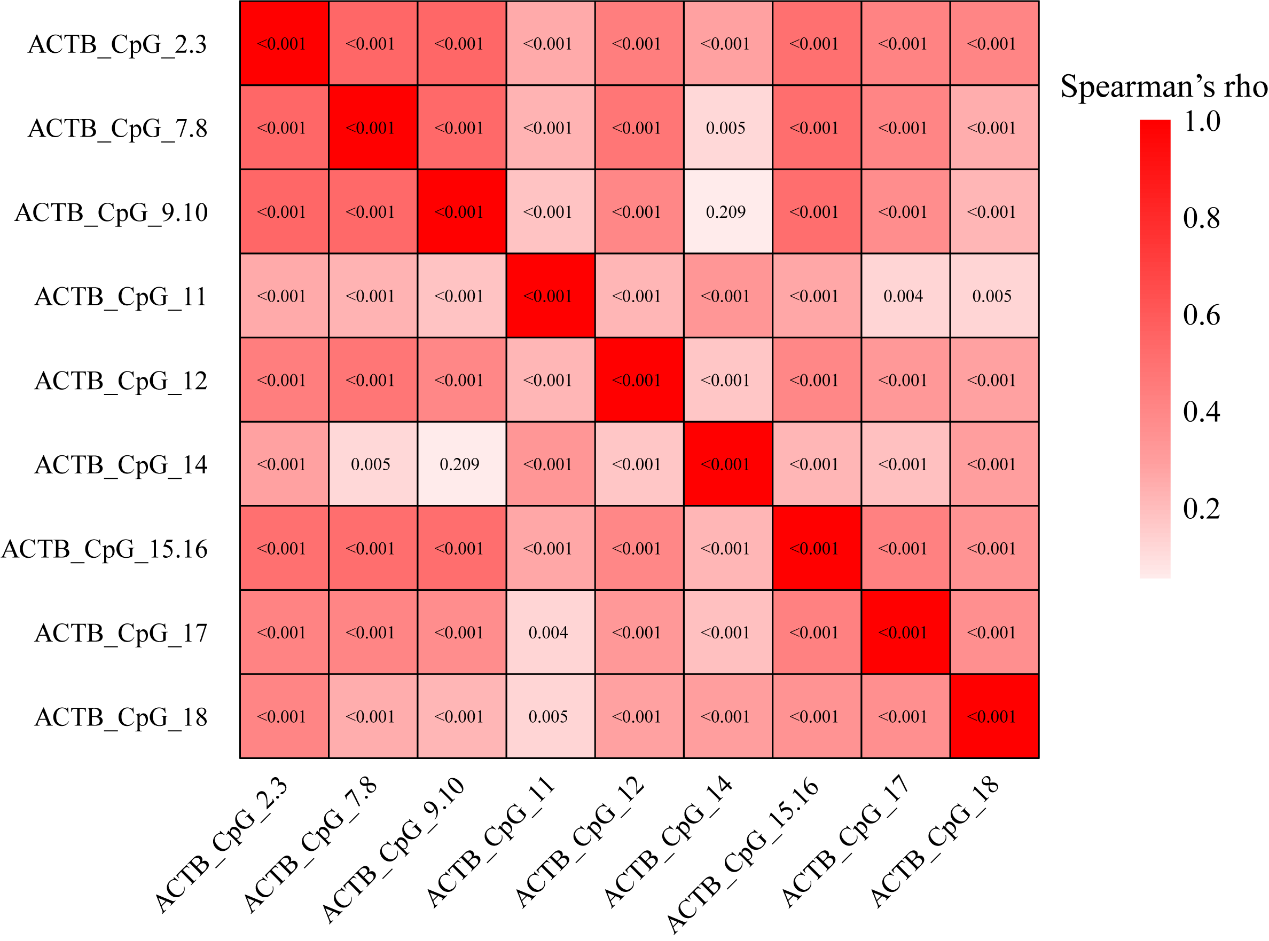


**Supplementary Figure 1.** Correlations among the methylation levels of the CpG sites in the *ACTB* amplicon. Heatmap showing *ACTB* CpG unit inter-correlations. The positively correlated CpG sites are in red. The color bar represents the strength of correlation as Spearman’s rho. *p*-values of correlations are indicated for each CpG unit pair in the correspondent box.
