# Supplementary material for: The association between ACTB methylation in peripheral blood and coronary heart disease in a case-control study
Source: Front Cardiovasc Med. 2022 Aug 18;9:972566. doi: 10.3389/fcvm.2022.972566 (PMC9433772; doi:10.3389/fcvm.2022.972566)
Supplement: Supplementary file 2 [file Data_Sheet_2.docx]

**Supplementary Tables**

**Supplementary Table 1.** The bisulfite specific primers of *ACTB*

| Primer | Sequences |
| --- | --- |
| ACTB-F | aggaagagagGGGATTTGATTGATTATTTTATGAAGA |
| ACTB-R | cagtaatacgactcactatagggagaaggctACCACAAAACTCCATACCTAAAAAA |
| Note: upper case letters present the sequence specific primer regions, and non-specific tags are shown in lower case letters. | |

| **Supplementary Table 2.** The reproducibility of mass spectrometry | | | | | | | | | | | |
| --- | --- | --- | --- | --- | --- | --- | --- | --- | --- | --- | --- |
| **CpG sites** | **ACTB_CpG**  **_2.3** | **ACTB_CpG**  **_4.5** | **ACTB_CpG**  **_6** | **ACTB_CpG**  **_7.8** | **ACTB_CpG**  **_9.10** | **ACTB_CpG**  **_11** | **ACTB_CpG**  **_12** | **ACTB_CpG**  **_14** | **ACTB_CpG**  **_15.16** | **ACTB_CpG**  **_17** | **ACTB_CpG**  **_18** |
| **Methylation levels** | 0.42 | 0.16 | 0.59 | 0.37 | 0.25 | 0.25 | 0.31 | 0.65 | 0.48 | 0.30 | 0.24 |
|  | 0.48 | 0.24 | 0.59 | 0.36 | 0.32 | 0.30 | 0.28 | 0.70 | 0.52 | 0.36 | 0.26 |
|  | 0.52 | 0.40 | 0.73 | 0.42 | 0.35 | 0.36 | 0.25 | 0.71 | 0.45 | 0.33 | 0.26 |
|  | 0.54 | 0.35 | 0.84 | 0.45 | 0.27 | 0.24 | 0.23 | 0.70 | 0.55 | 0.35 | 0.31 |
|  | 0.44 | 0.27 | 0.68 | 0.36 | 0.24 | 0.29 | 0.18 | 0.60 | 0.45 | 0.34 | 0.23 |
| **Standard deviation** | 0.050 | 0.094 | 0.105 | 0.041 | 0.047 | 0.048 | 0.050 | 0.047 | 0.044 | 0.023 | 0.031 |
| A randomly selected DNA sample was measured by mass spectrometry for five times. | | | | | | | | | | | |

| **Supplementary Table 3.** Distribution of *ACTB* factor loadings | | |
| --- | --- | --- |
|  | **Factor 1** | **Factor 2** |
| ACTB_CpG_2.3 | *0.823* | -0.027 |
| ACTB_CpG_15.16 | *0.807* | 0.060 |
| ACTB_CpG_7.8 | *0.713* | 0.354 |
| ACTB_CpG_17 | *0.682* | 0.306 |
| ACTB_CpG_12 | *0.623* | 0.249 |
| ACTB_CpG_9.10 | *0.525* | 0.328 |
| ACTB_CpG_14 | 0.005 | *0.859* |
| ACTB_CpG_11 | 0.174 | *0.586* |
| ACTB_CpG_18 | 0.275 | *0.561* |
| PCA resulted in the identification of two factors with eigenvalue > 1. Factor loadings of the main sites for each factor are highlighted in italics. | | |

| **Supplementary Table 4.** Association between *ACTB* methylation factors and CHD | | | | | |
| --- | --- | --- | --- | --- | --- |
| **Methylation factors** | **Model 1^a^** | |  | **Model 2^b^** | |
|  | **OR (95%CI) per +10% methylation** | ***p*-value** |  | **OR (95%CI) per +10% methylation** | ***p*-value** |
| Factor 1 | 1.74(1.43-2.12) | **3.49E-08** |  | 1.66(1.33-2.06) | **7.00E-06** |
| Factor 2 | 0.88(0.72-1.08) | 0.221 |  | 0.81(0.65-1.02) | 0.071 |
| ^a^Model 1: Logistic regression adjusted for age, gender, and batch.  ^b^Model 2: Logistic regression adjusted for age, gender, smoking, hypertension, diabetes, TC, TG, LDL-C, and batch effect. Significant *p*-values are in bold. | | | | | |

| **Supplementary Table 5.** Gender-specific association between *ACTB* methylation factors and CHD | | | | | |
| --- | --- | --- | --- | --- | --- |
| **Methylation factors** | **Model 1^a^** | |  | **Model 2^b^** | |
|  | **OR (95%CI) per +10% methylation** | ***p*-value** |  | **OR (95%CI) per +10% methylation** | ***p*-value** |
| **A. Female** | | | | | |
| Factor 1 | 1.78(1.27-2.49) | **0.001** |  | 1.79(1.20-2.66) | **0.004** |
| Factor 2 | 0.84(0.57-1.23) | 0.361 |  | 0.72(0.47-1.12) | 0.148 |
| **B. Male** | | | | | |
| Factor 1 | 1.72(1.35-2.19) | **1.30E-05** |  | 1.63(1.24-2.14) | **4.42E-04** |
| Factor 2 | 0.88(0.69-1.12) | 0.303 |  | 0.85(0.65-1.12) | 0.242 |
| ^a^Model 1: Logistic regression adjusted for age, gender, and batch.  ^b^Model 2: Logistic regression adjusted for age, gender, smoking, hypertension, diabetes, TC, TG, LDL-C, and batch effect. Significant *p*-values are in bold. | | | | | |

| **Supplementary Table 6.** Age-specific association between *ACTB* methylation factors and CHD | | | | | |
| --- | --- | --- | --- | --- | --- |
| **Methylation factors** | **Model 1^a^** | |  | **Model 2^b^** | |
|  | **OR (95%CI) per +10% methylation** | ***p*-value** |  | **OR (95%CI) per +10% methylation** | ***p*-value** |
| **A. Age < 60 years** | | | | | |
| Factor 1 | 1.87(1.43-2.45) | **5.00E-06** |  | 1.70(1.24-2.32) | **0.001** |
| Factor 2 | 0.83(0.65-1.08) | 0.163 |  | 0.77(0.57-1.05) | 0.097 |
| **B. Age ≥ 60 years** | | | | | |
| Factor 1 | 1.71(1.26-2.32) | **0.001** |  | 1.79(1.27-2.51) | **0.001** |
| Factor 2 | 0.93(0.66-1.32) | 0.701 |  | 0.83(0.57-1.21) | 0.341 |
| ^a^Model 1: Logistic regression adjusted for age, gender, and batch.  ^b^Model 2: Logistic regression adjusted for age, gender, smoking, hypertension, diabetes, TC, TG, LDL-C, and batch effect. Significant *p*-values are in bold. | | | | | |

| **Supplementary Table 7.** Association between *ACTB* methylation factors and MI | | | | | |
| --- | --- | --- | --- | --- | --- |
| **Methylation factors** | **Model 1^a^** | |  | **Model 2^b^** | |
|  | **OR (95%CI) per +10% methylation** | ***p*-value** |  | **OR (95%CI) per +10% methylation** | ***p*-value** |
| **A. 206 non-MI CHD cases vs. 272 controls** | | | | | |
| Factor 1 | 1.75(1.41-2.18) | **4.11E-07** |  | 1.64(1.28-2.09) | **7.50E-05** |
| Factor 2 | 1.12(0.89-1.41) | 0.353 |  | 1.04(0.80-1.35) | 0.771 |
| **B. 75 MI CHD cases vs. 272 controls** | | | | | |
| Factor 1 | 1.72(1.27-2.32) | **4.47E-04** |  | 1.68(1.22-2.33) | **0.002** |
| Factor 2 | 0.45(0.31-0.66) | **3.60E-05** |  | 0.43(0.29-0.64) | **4.20E-05** |
| ^a^Model 1: Logistic regression adjusted for age, gender, and batch.  ^b^Model 2: Logistic regression adjusted for age, gender, smoking, hypertension, diabetes, TC, TG, LDL-C, and batch effect. Significant *p*-values are in bold. | | | | | |

**Supplementary Table 8.** Methylation difference of *ACTB* between non-HF CHD cases and controls

| **CpG sites** | **Controls (N = 272)** | **Non-HF CHD cases (N = 84)** | **Model 1^a^** | |  | **Model 2^b^** | |
| --- | --- | --- | --- | --- | --- | --- | --- |
|  | **Median (IQR)** | **Median (IQR)** | **OR (95%CI) per +10%methylation** | ***p-*value** |  | **OR (95%CI) per +10%methylation** | ***p-*value** |
| ACTB_CpG_2.3 | 0.48(0.40-0.57) | 0.57(0.44-0.68) | 1.40(1.16-1.70) | **4.79E-04** |  | 1.28(1.03-1.59) | **0.024** |
| ACTB_CpG_7.8 | 0.35(0.27-0.42) | 0.38(0.29-0.49) | 1.32(1.09-1.59) | **0.005** |  | 1.14(0.92-1.42) | 0.235 |
| ACTB_CpG_9.10 | 0.30(0.23-0.36) | 0.34(0.27-0.43) | 1.48(1.17-1.87) | **0.001** |  | 1.33(1.02-1.73) | **0.034** |
| ACTB_CpG_11 | 0.70(0.61-0.81) | 0.71(0.60-0.81) | 0.97(0.82-1.13) | 0.678 |  | 0.90(0.75-1.08) | 0.245 |
| ACTB_CpG_12 | 0.25(0.17-0.33) | 0.29(0.20-0.39) | 1.34(1.11-1.63) | **0.003** |  | 1.32(1.06-1.64) | **0.015** |
| ACTB_CpG_14 | 0.54(0.43-0.72) | 0.65(0.45-0.88) | 0.96(0.84-1.10) | 0.550 |  | 0.91(0.78-1.07) | 0.253 |
| ACTB_CpG_15.16 | 0.53(0.42-0.62) | 0.61(0.51-0.69) | 1.38(1.15-1.66) | **0.001** |  | 1.31(1.06-1.61) | **0.011** |
| ACTB_CpG_17 | 0.33(0.26-0.40) | 0.35(0.28-0.48) | 1.12(0.95-1.32) | 0.185 |  | 1.12(0.93-1.35) | 0.245 |
| ACTB_CpG_18 | 0.32(0.25-0.39) | 0.33(0.26-0.43) | 1.01(0.86-1.20) | 0.876 |  | 1.03(0.86-1.24) | 0.739 |
| ^a^Model 1: Logistic regression adjusted for age, gender, and batch.  ^b^Model 2: Logistic regression adjusted for age, gender, smoking, hypertension, diabetes, TC, TG, LDL-C, and batch effect. Significant *p*-values are in bold. | | | | | | | |

| **Supplementary Table 9.** Methylation difference of *ACTB* between different types of HF cases and controls | | | | | |
| --- | --- | --- | --- | --- | --- |
| **CpG sites** | **Controls (N=272)** | **HFpEF (N=156)** | **HFmrEF (N=15)** | **HFrEF (N=26)** | ***p*-value** |
|  | **Median (IQR)** | **Median (IQR)** | **Median (IQR)** | **Median (IQR)** |  |
| ACTB_CpG_2.3 | 0.48(0.40-0.57) | 0.54(0.47-0.63) | 0.50(0.41-0.57) | 0.62(0.43-0.74) | **1.60E-05** |
| ACTB_CpG_7.8 | 0.35(0.27-0.42) | 0.40(0.32-0.49) | 0.39(0.27-0.51) | 0.46(0.27-0.58) | **1.85E-04** |
| ACTB_CpG_9.10 | 0.30(0.23-0.36) | 0.36(0.29-0.42) | 0.32(0.25-0.44) | 0.37(0.30-0.45) | **3.00E-06** |
| ACTB_CpG_11 | 0.70(0.61-0.81) | 0.71(0.59-0.80) | 0.62(0.50-0.70) | 0.73(0.55-0.88) | 0.223 |
| ACTB_CpG_12 | 0.25(0.17-0.33) | 0.25(0.17-0.35) | 0.24(0.20-0.30) | 0.26(0.15-0.35) | 0.720 |
| ACTB_CpG_14 | 0.54(0.43-0.72) | 0.51(0.38-0.62) | 0.35(0.28-0.48) | 0.47(0.27-0.97) | **0.005** |
| ACTB_CpG_15.16 | 0.53(0.42-0.62) | 0.57(0.48-0.64) | 0.55(0.47-0.71) | 0.55(0.40-0.73) | **0.044** |
| ACTB_CpG_17 | 0.33(0.26-0.40) | 0.33(0.27-0.42) | 0.33(0.29-0.44) | 0.32(0.25-0.47) | 0.824 |
| ACTB_CpG_18 | 0.32(0.25-0.39) | 0.34(0.27-0.41) | 0.30(0.24-0.35) | 0.29(0.11-0.40) | 0.079 |
| Abbreviations: EF:ejection fraction; HFpEF: HF with preserved EF; HFmrEF: HF with mildly reduced EF; HFrEF: HF with reduced EF. | | | | | |
| Kruskal-Wallis test for methylation differences between groups. Significant *p*-values are in bold. | | | | | |

| **Supplementary Table 10.** Association between *ACTB* methylation factors and HF | | | | | |
| --- | --- | --- | --- | --- | --- |
| **Methylation factors** | **Model 1^a^** | |  | **Model 2^b^** | |
|  | **OR (95%CI) per +10% methylation** | ***p*-value** |  | **OR (95%CI) per +10% methylation** | ***p*-value** |
| **A. 84 non-HF CHD cases vs. 272 controls** | | | | | |
| Factor 1 | 1.74(1.30-2.33) | **2.11E-04** |  | 1.52(1.08-2.13) | **0.017** |
| Factor 2 | 0.87(0.63-1.21) | 0.405 |  | 0.81(0.56-1.17) | 0.258 |
| **B. 197 HF CHD cases vs. 272 controls** | | | | | |
| Factor 1 | 1.75(1.41-2.17) | **5.23E-07** |  | 1.69(1.33-2.14) | **1.50E-05** |
| Factor 2 | 0.85(0.68-1.07) | 0.176 |  | 0.79(0.62-1.02) | 0.071 |
| ^a^Model 1: Logistic regression adjusted for age, gender, and batch.  ^b^Model 2: Logistic regression adjusted for age, gender, smoking, hypertension, diabetes, TC, TG, LDL-C, and batch effect. Significant *p*-values are in bold. | | | | | |

| **Supplementary Table 11.** Association between *ACTB* methylation factors and NYHA Ⅰ&Ⅱ CHD cases | | | | | |
| --- | --- | --- | --- | --- | --- |
| **Methylation factors** | **Model 1^a^** | |  | **Model 2^b^** | |
|  | **OR (95%CI) per +10% methylation** | ***p*-value** |  | **OR (95%CI) per +10% methylation** | ***p*-value** |
| Factor 1 | 1.78(1.40-2.27) | **3.00E-06** |  | 1.67(1.28-2.18) | **1.37E-04** |
| Factor 2 | 0.90(0.70-1.17) | 0.445 |  | 0.82(0.61-1.09) | 0.168 |
| ^a^Model 1: Logistic regression adjusted for age, gender, and batch.  ^b^Model 2: Logistic regression adjusted for age, gender, smoking, hypertension, diabetes, TC, TG, LDL-C, and batch effect. Significant *p*-values are in bold. | | | | | |

**Supplementary Table 12.** The correlation between *ACTB* methylation and the clinical characteristics of participants

| **Correlation with HDL-C** | | | | |
| --- | --- | --- | --- | --- |
| CpG sites | Controls (N = 272) | | CHD cases (N = 281) | |
|  | r | *p-*value^a^ | r | *p-*value^a^ |
| ACTB_CpG_2.3 | -0.106 | 0.090 | -0.124 | **0.039** |
| ACTB_CpG_7.8 | -0.030 | 0.630 | -0.226 | **1.45E-04** |
| ACTB_CpG_9.10 | -0.140 | **0.023** | -0.048 | 0.424 |
| ACTB_CpG_11 | -0.071 | 0.251 | -0.064 | 0.286 |
| ACTB_CpG_12 | -0.079 | 0.200 | -0.132 | **0.027** |
| ACTB_CpG_14 | -0.095 | 0.124 | -0.040 | 0.504 |
| ACTB_CpG_15.16 | -0.117 | 0.057 | -0.068 | 0.259 |
| ACTB_CpG_17 | -0.057 | 0.357 | -0.112 | 0.063 |
| ACTB_CpG_18 | -0.109 | 0.079 | -0.064 | 0.289 |
| **Correlation with LDL-C** | | | | |
| CpG sites | Controls (N = 272) | | CHD cases (N = 281) | |
|  | r | *p-*value^a^ | r | *p-*value^a^ |
| ACTB_CpG_2.3 | -0.018 | 0.779 | -0.069 | 0.253 |
| ACTB_CpG_7.8 | -0.027 | 0.664 | -0.022 | 0.710 |
| ACTB_CpG_9.10 | -0.063 | 0.308 | -0.070 | 0.241 |
| ACTB_CpG_11 | 0.038 | 0.538 | -0.054 | 0.363 |
| ACTB_CpG_12 | -0.057 | 0.353 | -0.003 | 0.963 |
| ACTB_CpG_14 | 0.025 | 0.690 | 0.004 | 0.952 |
| ACTB_CpG_15.16 | -0.089 | 0.151 | -0.005 | 0.938 |
| ACTB_CpG_17 | -0.075 | 0.225 | 0.053 | 0.376 |
| ACTB_CpG_18 | -0.034 | 0.585 | -0.058 | 0.338 |
| **Correlation with TC** | | | | |
| CpG sites | Controls (N = 272) | | CHD cases (N = 281) | |
|  | r | *p-*value^a^ | r | *p-*value^a^ |
| ACTB_CpG_2.3 | -0.075 | 0.228 | -0.095 | 0.113 |
| ACTB_CpG_7.8 | -0.066 | 0.286 | -0.049 | 0.416 |
| ACTB_CpG_9.10 | -0.116 | 0.060 | -0.052 | 0.390 |
| ACTB_CpG_11 | 0.010 | 0.868 | -0.035 | 0.565 |
| ACTB_CpG_12 | -0.117 | 0.058 | -0.016 | 0.787 |
| ACTB_CpG_14 | -0.011 | 0.855 | -0.014 | 0.815 |
| ACTB_CpG_15.16 | -0.143 | **0.020** | 0.003 | 0.956 |
| ACTB_CpG_17 | -0.100 | 0.104 | 0.025 | 0.675 |
| ACTB_CpG_18 | -0.076 | 0.222 | -0.059 | 0.328 |
| **Correlation with TG** | | | | |
| CpG sites | Controls (N = 272) | | CHD cases (N = 281) | |
|  | r | *p-*value^a^ | r | *p-*value^a^ |
| ACTB_CpG_2.3 | -0.035 | 0.573 | 0.075 | 0.212 |
| ACTB_CpG_7.8 | -0.121 | 0.050 | 0.129 | **0.032** |
| ACTB_CpG_9.10 | -0.049 | 0.425 | 0.057 | 0.345 |
| ACTB_CpG_11 | 0.038 | 0.538 | -0.021 | 0.731 |
| ACTB_CpG_12 | -0.091 | 0.142 | 0.091 | 0.130 |
| ACTB_CpG_14 | 0.019 | 0.761 | 0.037 | 0.539 |
| ACTB_CpG_15.16 | -0.059 | 0.341 | 0.075 | 0.214 |
| ACTB_CpG_17 | -0.036 | 0.562 | 0.064 | 0.289 |
| ACTB_CpG_18 | -0.082 | 0.186 | 0.014 | 0.820 |
| **Correlation with smoking** | | | | |
| CpG sites | Controls (N = 272) | | CHD cases (N = 281) | |
|  | C | *p-*value^b^ | C | *p-*value^b^ |
| ACTB_CpG_2.3 | 0.447 | 0.478 | 0.464 | 0.270 |
| ACTB_CpG_7.8 | 0.428 | 0.381 | 0.427 | 0.633 |
| ACTB_CpG_9.10 | 0.440 | 0.100 | 0.399 | 0.582 |
| ACTB_CpG_11 | 0.406 | 0.793 | 0.420 | 0.576 |
| ACTB_CpG_12 | 0.433 | 0.221 | 0.448 | 0.169 |
| ACTB_CpG_14 | 0.469 | 0.531 | 0.440 | 0.838 |
| ACTB_CpG_15.16 | 0.464 | 0.313 | 0.418 | 0.658 |
| ACTB_CpG_17 | 0.453 | 0.208 | 0.476 | 0.129 |
| ACTB_CpG_18 | 0.415 | 0.519 | 0.434 | 0.638 |
| **Correlation with drinking** | | | | |
| CpG sites | Controls (N = 272) | | CHD cases (N = 281) | |
|  | C | *p-*value^b^ | C | *p-*value^b^ |
| ACTB_CpG_2.3 | 0.446 | 0.489 | 0.440 | 0.573 |
| ACTB_CpG_7.8 | 0.386 | 0.836 | 0.406 | 0.849 |
| ACTB_CpG_9.10 | 0.445 | 0.076 | 0.360 | 0.920 |
| ACTB_CpG_11 | 0.435 | 0.458 | 0.462 | 0.124 |
| ACTB_CpG_12 | 0.415 | 0.411 | 0.420 | 0.464 |
| ACTB_CpG_14 | 0.517 | 0.061 | 0.453 | 0.712 |
| ACTB_CpG_15.16 | 0.473 | 0.215 | 0.450 | 0.267 |
| ACTB_CpG_17 | 0.437 | 0.374 | 0.455 | 0.334 |
| ACTB_CpG_18 | 0.397 | 0.723 | 0.458 | 0.337 |
| **Correlation with hypertension** | | | | |
| CpG sites | Controls (N = 272) | | CHD cases (N = 281) | |
|  | C | *p-*value^b^ | C | *p-*value^b^ |
| ACTB_CpG_2.3 | 0.427 | 0.672 | 0.450 | 0.442 |
| ACTB_CpG_7.8 | 0.406 | 0.690 | 0.431 | 0.581 |
| ACTB_CpG_9.10 | 0.410 | 0.434 | 0.407 | 0.482 |
| ACTB_CpG_11 | 0.435 | 0.518 | 0.448 | 0.235 |
| ACTB_CpG_12 | 0.402 | 0.573 | 0.387 | 0.833 |
| ACTB_CpG_14 | 0.491 | 0.309 | 0.517 | **0.046** |
| ACTB_CpG_15.16 | 0.412 | 0.869 | 0.437 | 0.419 |
| ACTB_CpG_17 | 0.432 | 0.483 | 0.471 | 0.171 |
| ACTB_CpG_18 | 0.451 | 0.175 | 0.412 | 0.855 |
| **Correlation with diabetes** | | | | |
|  | | | | |
| CpG sites | Controls (N = 272) | | CHD cases (N = 281) | |
|  | C | *p-*value^b^ | C | *p-*value^b^ |
| ACTB_CpG_2.3 | 0.458 | 0.301 | 0.458 | 0.340 |
| ACTB_CpG_7.8 | 0.419 | 0.553 | 0.406 | 0.844 |
| ACTB_CpG_9.10 | 0.387 | 0.711 | 0.396 | 0.621 |
| ACTB_CpG_11 | 0.472 | 0.130 | 0.384 | 0.909 |
| ACTB_CpG_12 | 0.364 | 0.907 | 0.425 | 0.407 |
| ACTB_CpG_14 | 0.474 | 0.539 | 0.457 | 0.658 |
| ACTB_CpG_15.16 | 0.389 | 0.968 | 0.451 | 0.258 |
| ACTB_CpG_17 | 0.413 | 0.747 | 0.444 | 0.475 |
| ACTB_CpG_18 | 0.467 | 0.082 | 0.462 | 0.283 |
| ^a^The *p*-values were calculated by Spearman rank test method.  ^b^The *p*-values were calculated by contingency coefficient correlation method, and significant *p*-values are in bold. | | | | |

**Supplementary Table 13.** The methylation of *ACTB* in CHD patients with various medical treatments

| **Medicine** | **Group (N)** | **Median of methylation intensity** | | | | | | | | |
| --- | --- | --- | --- | --- | --- | --- | --- | --- | --- | --- |
|  |  | **ACTB_CpG**  **_2.3** | **ACTB_CpG**  **_7.8** | **ACTB_CpG**  **_9.10** | **ACTB_CpG**  **_11** | **ACTB_CpG**  **_12** | **ACTB_CpG**  **_14** | **ACTB_CpG**  **_15.16** | **ACTB_CpG**  **_17** | **ACTB_CpG**  **_18** |
| **β blocker** | No (104) | 0.53 | 0.39 | 0.33 | 0.68 | 0.25 | 0.49 | 0.57 | 0.33 | 0.32 |
|  | Yes (177) | 0.56 | 0.40 | 0.37 | 0.72 | 0.28 | 0.54 | 0.57 | 0.35 | 0.34 |
|  | *p*-value^*^ | **0.014** | 0.357 | **0.005** | **0.006** | **0.023** | 0.097 | 0.232 | 0.300 | 0.267 |
| **Digoxin** | No (267) | 0.54 | 0.39 | 0.36 | 0.70 | 0.26 | 0.53 | 0.57 | 0.34 | 0.33 |
|  | Yes (14) | 0.57 | 0.49 | 0.37 | 0.82 | 0.29 | 0.50 | 0.65 | 0.34 | 0.33 |
|  | *p*-value^*^ | 0.389 | **0.026** | 0.564 | **0.047** | 0.133 | 0.818 | **0.044** | 0.662 | 0.830 |
| **Aspirin** | No (63) | 0.53 | 0.39 | 0.32 | 0.66 | 0.23 | 0.48 | 0.55 | 0.33 | 0.33 |
|  | Yes (218) | 0.55 | 0.40 | 0.36 | 0.71 | 0.28 | 0.54 | 0.58 | 0.35 | 0.34 |
|  | *p*-value^*^ | 0.143 | 0.456 | 0.198 | **0.007** | **0.031** | 0.113 | 0.533 | 0.275 | 0.532 |
| **Statin** | No (39) | 0.53 | 0.36 | 0.34 | 0.69 | 0.21 | 0.48 | 0.52 | 0.31 | 0.33 |
|  | Yes (242) | 0.55 | 0.40 | 0.36 | 0.70 | 0.28 | 0.53 | 0.59 | 0.35 | 0.34 |
|  | *p*-value^*^ | 0.266 | 0.366 | 0.466 | 0.343 | **0.009** | 0.934 | 0.052 | 0.069 | 0.571 |
| **ACEI** | No (242) | 0.54 | 0.40 | 0.36 | 0.71 | 0.27 | 0.53 | 0.57 | 0.34 | 0.33 |
|  | Yes (39) | 0.52 | 0.40 | 0.36 | 0.66 | 0.26 | 0.52 | 0.58 | 0.35 | 0.34 |
|  | *p*-value^*^ | 0.977 | 0.374 | 0.733 | 0.589 | 0.492 | 0.218 | 0.509 | 0.802 | 0.521 |
| **ARB** | No (203) | 0.55 | 0.40 | 0.37 | 0.70 | 0.27 | 0.53 | 0.58 | 0.34 | 0.34 |
|  | Yes (78) | 0.54 | 0.39 | 0.33 | 0.71 | 0.27 | 0.55 | 0.57 | 0.35 | 0.31 |
|  | *p*-value^*^ | 0.643 | 0.318 | 0.086 | 0.777 | 0.316 | 0.337 | 0.206 | 0.273 | 0.185 |
| **CCB** | No (198) | 0.55 | 0.40 | 0.36 | 0.71 | 0.27 | 0.52 | 0.57 | 0.35 | 0.33 |
|  | Yes (83) | 0.54 | 0.38 | 0.36 | 0.67 | 0.26 | 0.54 | 0.57 | 0.33 | 0.32 |
|  | *p*-value^*^ | 0.623 | 0.104 | 0.912 | 0.088 | 0.590 | 0.698 | 0.860 | 0.641 | 0.625 |
| **Spironolactone** | No (232) | 0.55 | 0.39 | 0.36 | 0.70 | 0.27 | 0.53 | 0.58 | 0.34 | 0.34 |
|  | Yes (49) | 0.53 | 0.45 | 0.34 | 0.74 | 0.28 | 0.56 | 0.57 | 0.35 | 0.33 |
|  | *p*-value^*^ | 0.592 | 0.065 | 0.904 | 0.410 | 0.492 | 0.733 | 0.785 | 0.790 | 0.207 |
| **Nitrates** | No (147) | 0.55 | 0.39 | 0.35 | 0.70 | 0.26 | 0.53 | 0.58 | 0.34 | 0.33 |
|  | Yes (134) | 0.54 | 0.40 | 0.37 | 0.71 | 0.29 | 0.53 | 0.57 | 0.33 | 0.34 |
|  | *p*-value^*^ | 0.668 | 0.662 | 0.936 | 0.585 | 0.050 | 0.660 | 0.771 | 0.285 | 0.540 |
| **Clopidogrel** | No (135) | 0.55 | 0.40 | 0.36 | 0.69 | 0.25 | 0.54 | 0.57 | 0.36 | 0.34 |
|  | Yes (145) | 0.54 | 0.39 | 0.35 | 0.72 | 0.27 | 0.53 | 0.58 | 0.33 | 0.33 |
|  | *p*-value^*^ | 0.911 | 0.452 | 0.959 | 0.070 | 0.157 | 0.579 | 0.535 | 0.205 | 0.748 |
| **Warfarin** | No (271) | 0.55 | 0.40 | 0.36 | 0.70 | 0.27 | 0.53 | 0.57 | 0.34 | 0.33 |
|  | Yes (10) | 0.51 | 0.37 | 0.31 | 0.65 | 0.22 | 0.51 | 0.55 | 0.33 | 0.38 |
|  | *p*-value^*^ | 0.205 | 0.822 | 0.415 | 0.103 | 0.714 | 0.712 | 0.651 | 0.448 | 0.887 |
| **Antacids** | No (187) | 0.54 | 0.40 | 0.36 | 0.70 | 0.26 | 0.53 | 0.59 | 0.34 | 0.33 |
|  | Yes (94) | 0.54 | 0.39 | 0.34 | 0.70 | 0.29 | 0.53 | 0.57 | 0.33 | 0.34 |
|  | *p*-value^*^ | 0.798 | 0.656 | 0.113 | 0.756 | 0.237 | 0.821 | 0.441 | 0.966 | 0.842 |
| Abbreviations: ACEI: angiotensin converting enzyme inhibitor; ARB: angiotensin receptor blocker; CCB: calcium channel blockers.  ^*^The *p*-values were calculated by the Mann-Whitney test, and significant *p*-values are in bold. | | | | | | | | | | |

**Supplementary Table 14.** The discriminatory power of *ACTB* methylation to distinguish CHD cases from controls

| **All 281 CHD cases vs. all 272 controls** | | | |
| --- | --- | --- | --- |
| **CpG sites** | **AUC, 95% CI^a^** |  | **AUC, 95% CI^b^** |
| ACTB_CpG_2.3&ACTB_CpG_7.8&ACTB_CpG_9.10 | 0.67(0.63-0.72) |  | 0.75(0.70-0.79) |
| All *ACTB* CpG sites | 0.68(0.63-0.72) |  | 0.75(0.71-0.79) |
| **180 CHD cases vs. 182 controls, male** | | | |
| **CpG sites** | **AUC, 95% CI^a^** |  | **AUC, 95% CI^b^** |
| ACTB_CpG_2.3&ACTB_CpG_7.8&ACTB_CpG_9.10 | 0.66(0.60-0.71) |  | 0.74(0.68-0.79) |
| All *ACTB* CpG sites | 0.65(0.60-0.71) |  | 0.74(0.68-0.79) |
| **197 HF cases vs. all 272 controls** | | | |
| **CpG sites** | **AUC, 95% CI^a^** |  | **AUC, 95% CI^b^** |
| ACTB_CpG_2.3&ACTB_CpG_7.8&ACTB_CpG_9.10 | 0.68(0.63-0.73) |  | 0.73(0.69-0.78) |
| All *ACTB* CpG sites | 0.70(0.65-0.75) |  | 0.74(0.69-0.78) |
| **153 NYHA Ⅰ&Ⅱ CHD cases vs. all 272 controls** | | | |
| **CpG sites** | **AUC, 95% CI^a^** |  | **AUC, 95% CI^b^** |
| ACTB_CpG_2.3&ACTB_CpG_7.8&ACTB_CpG_9.10 | 0.70(0.64-0.75) |  | 0.77(0.72-0.81) |
| All *ACTB* CpG sites | 0.71(0.65-0.76) |  | 0.76(0.71-0.81) |
| Model a: Logistic regression adjusted for age, gender, and batch effect.  Model b: Logistic regression adjusted for age, gender, smoking, hypertension, diabetes, TC, TG, LDL-C, and batch effect. | | | |
